# Disparate developmental patterns of immune responses to bacterial and viral infections in fish

**DOI:** 10.1038/srep15458

**Published:** 2015-10-21

**Authors:** Rosario Castro, Luc Jouneau, Luca Tacchi, Daniel J. Macqueen, Abdullah Alzaid, Christopher J. Secombes, Samuel A. M. Martin, Pierre Boudinot

**Affiliations:** 1 Virologie et Immunologie Moléculaires, INRA, Jouy-en-Josas, France; 2 Institute of Biological and Environmental Sciences, University of Aberdeen, Aberdeen, United Kingdom, AB24 2TZ

## Abstract

During early stages of development vertebrates rely on an immature immune system to fight pathogens, but in non mammalian species few studies have taken an in-depth analysis of the transition from reliance on innate immune mechanisms to the appearance of adaptive immunity. Using rainbow trout as a model we characterized responses to two natural pathogens of this species, the Gram negative bacterium *Aeromonas salmonicida* and the virus VHSV, using microarray analysis at four early life history stages; eyed egg, post hatch, first feeding and three weeks post first feeding when adaptive immunity starts to be effective. All stages responded to both infections, but the complexity of the response increased with developmental stage. The response to virus showed a clear interferon response only from first feeding. In contrast, bacterial infection induced a marked response from early stages, with modulation of inflammatory, antimicrobial peptide and complement genes across all developmental stages. Whilst the viral and bacterial responses were distinct, there were modulated genes in common, mainly of general inflammatory molecules. This work provides a first platform to explore the development of fish immunity to infection, and to compare the age-dependent changes (from embryo to adults) across vertebrates.

In most non mammalian vertebrates, pre- and post- hatch life occurs in an unpredictable, hostile external environment. This is in contrast to mammals where the embryo is in an almost sterile environment where pathogen exposure is controlled by the mother. Hence, fish eggs and embryos have to be able to fight efficiently against infections, and must possess active defence mechanisms.

In teleost eggs, the first line of defence against invading pathogens is the physical barrier of the chorion and membranes^1^,^2^. Additionally, the embryonic membranes may express molecules to prevent bacterial and viral invasion, and may lack specific receptors for viral entry. The eggs at the early stages are also protected by maternally derived immune factors that are incorporated during vitellogenesis^3^, including complement components and other antimicrobial factors^4^,^5^,^6^,^7^.

A number of critical transitions occur during embryo and fry development. Hatching is a first drastic change. Mouth and gut opening and first feeding stages represent another critical transition: as the gut microbiota is established^8^, interactions between the developing teleost immune system and the environment intensify, increasing dramatically the need for efficient defence mechanisms against pathogens. Importantly, lymphocytes and other components of adaptive immunity appear progressively and do not efficiently protect the fish until well after hatching.

In fish as in other vertebrates, innate immunity is particularly important in early phases of development, when adaptive immunity is still not (fully) established and functioning. Triggering of innate receptors specific for pathogen-specific molecular patterns activates a variety of signalling pathways. Generic defence mechanisms like proinflammatory responses lead to wide cell activation, but pathogen type-specific mechanisms like the type I interferon (IFN) response are also triggered. Essential players of this pattern recognition receptor (PRR) system are the Toll-like receptors (TLRs) and the retinoic acid-inducible gene I (RIGI)-like receptors (RLR)^9^,^10^,^11^,^12^. In the case of virus infection, specialized PRRs that recognize viral RNA or DNA nucleic acids are remarkably conserved between teleosts and mammals, indicating an early origin of antiviral innate immunity within vertebrates^13^,^14^,^15^ (reviewed in^16^). While such immune pathways are highly conserved, much remains to be discovered about how they are established during development, and participate in defence of the early juvenile stages in different species. In humans, distinct phases of the development of immunity have been distinguished based on levels of TLR-induced cytokine responses^17^. These periods are each associated with predominant infectious diseases reflecting the immaturity of responses. Whether such transitions during the development of immunity are conserved across vertebrates remains unknown.

Here we examine for the first time the divergence between responses to bacterial and viral infections during early development in a teleost fish. We exposed key development stages from eyed eggs to free feeding animals to two pathogenic models; a virus and a bacterial species. Transcriptomic profiling demonstrated bacterial-induced and common pathogen defences at early stages with a clearer specific response to the virus at the older stages. We found at the time of first feeding that components of the acquired immune system also become more responsive. The profiles substantially increase the understanding of complex molecular responses to pathogens, and also represent a valuable tool to aid improvement of disease control and vaccine development in aquaculture. There are common themes that appear conserved on an evolutionary scale with mammals, in addition to unique features in relation to early protection required for independent living.

## Results

### A massive transcriptome response to *A. salmonicida* takes place from the egg stage, while the response to VHSV matures progressively

**Figure 1:**
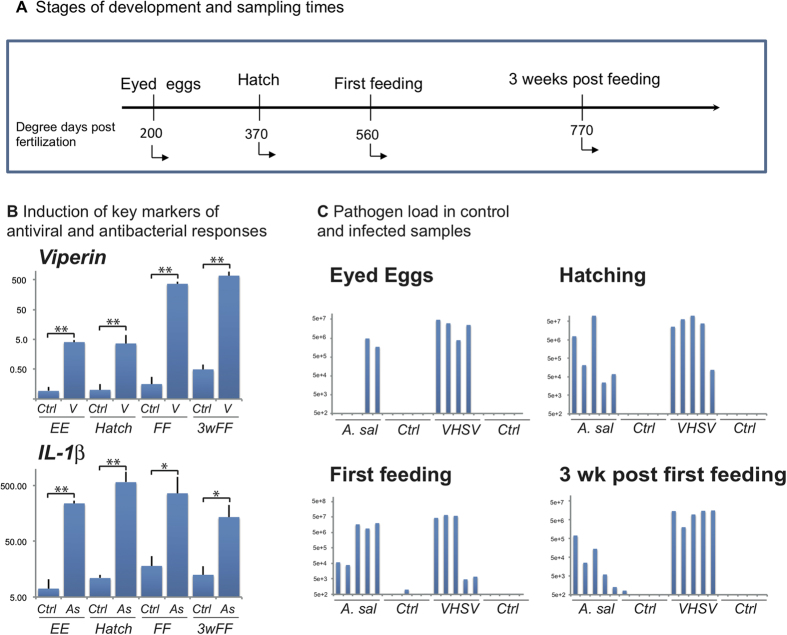
Design of the fish infection and transcriptome experiments. (**A**) Stages of development and sampling times. (**B**) Induction of viperin and IL-1β after viral and bacterial injection, respectively (mean ±SE; N = 4), at selected stages of development. Significant differences were determined using unpaired *t* test (*P < 0.05; **P < 0.001). (**C**) Pathogen load in control and infected individuals assessed by VHSV or *A. salmonicida* specific real time PCR assay. The efficiency of each qPCR assay was determined using LinRegPCR. Results are represented in arbitrary units.

Infections were performed in parallel either by bath or by injection with two pathogens (analysed individually), VHSV and *A. salmonicida*, for which comprehensive descriptions of infection-induced modifications of the transcriptome have been reported in adult tissues^16^,^18^. We examined four critical developmental stages of rainbow trout: eyed eggs (EE), hatching fry (H), alevins at first feeding (FF) and three weeks later (3wFF) (Fig. 1A). Fish were sampled three days post-infection, when we anticipated that the transcriptome response would be well developed, but no clinical signs of disease or lesions were detectable. This was before the onset of mortality, and hence the impact of the general physiological shock due to lesions associated with the terminal phase of the disease should therefore be limited. Pathogen-induced responses were first investigated by real time PCR of key markers including *viperin/rsad2* and *IL-1β*, these markers indicating either an induced antiviral response (*viperin*) or proinflammatory response to bacteria (*IL-1β*). No clear response could be detected after bath infection, indicating that eyed egg to fry stages are well-protected from natural infection. In contrast, the injection route of infection led to up-regulation of the marker genes (Fig. 1B) and to a significant pathogen load at all stages for both pathogens (Fig. 1C).

To perform a comprehensive analysis of the transcriptional response induced by injection of VHSV and *A. salmonicida*, we used a microarray platform enriched for immune related genes to identify genes differentially expressed between infected and uninfected control animals at each stage for each pathogen. In this way we were able to analyse the pathogen-specific response at each time and also examine how the different pathogens induced common or specific responses during ontological development (Table S1 and S2).

**Figure 2:**
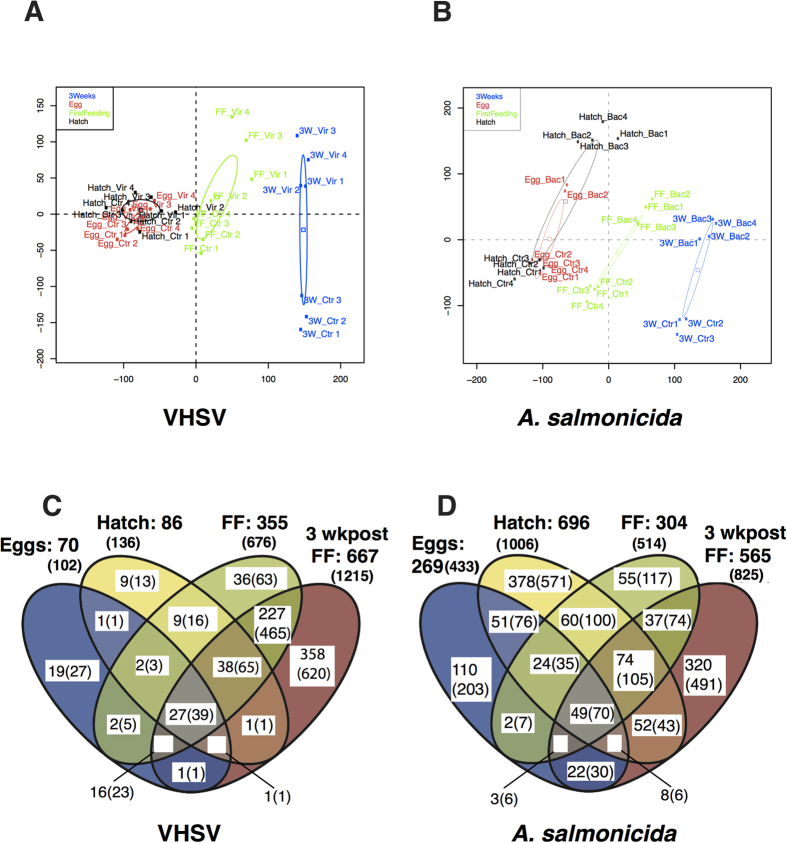
Global analysis of microarray data. (**A**) Principal component analysis of all features (except features deposited on the array for internal control) at four stages of development: eyed eggs “egg” in red; hatching “hatch” in black; first feeding “FF” in green and 3 weeks post first feeding “3w” in blue. Samples (pools of eggs or fry) are noted as either “vir” when infected by VHSV or “ctrl” for controls. Projection on the two first axis is shown (dimension 1: horizontal axis; dimension 2: vertical axis). (**B**) Similar principal component analysis of modulated features from samples infected by *A. salmonicida*, noted as “bac” or controls “ctrl”. (**C**,**D**) Venn diagrams showing the number of genes significantly up- or down- regulated at each stage of development (p < 0.01; Fold Change (FC) > 2 or <0.5) by VHSV or *A. salmonicida*. The corresponding numbers of array features are given in brackets.

To gain an overview of the global responses, principal component analysis (PCA) was separately performed with hybridization datasets for viral and bacterial infections (Fig. 2A,B). The transcriptomes were separated primarily by developmental stage as seen with dimension 1 (horizontal axis); both eyed eggs (EE) and hatch (H) stages were closely grouped, but were clearly different from first feeding (FF) and even more distant from 3 weeks post first feeding (3wFF). This observation was consistently observed for all fish groups (viral and bacterial infected fish, as well as controls). The second dimension (vertical axis) of the PCA represented differences between infected and non-infected fish, and revealed striking differences between the response to viral and bacterial infections (Fig. 2A,B). For the viral infection PCA did not reveal obvious differences between control and VHSV-infected fish at EE and H stages; in contrast, viral infected samples appeared well-separated at the FF stage, and a massive shift was observed at the 3wFF stage, thus suggesting a progressive maturation of virus-induced pathways across fry development. Regarding *A. salmonicida*, a completely different pattern was obtained, with a clear discrimination of infected and non-infected fish already at EE and H stages. Unlike the viral infection, the magnitude of the response at the FF and 3WFF did not appear to increase compared to earlier life stages.

The pattern we observed by PCA was supported by the number of significantly modulated genes (corrected *P*val < 1%, fold change >2 or <0.5) due to the infection at each stage (Fig. 2C,D). Together, these observations indicate that the maturation of pathways responsive to viral and bacterial infection does not occur in parallel during the first weeks of life of rainbow trout.

**Figure 3:**
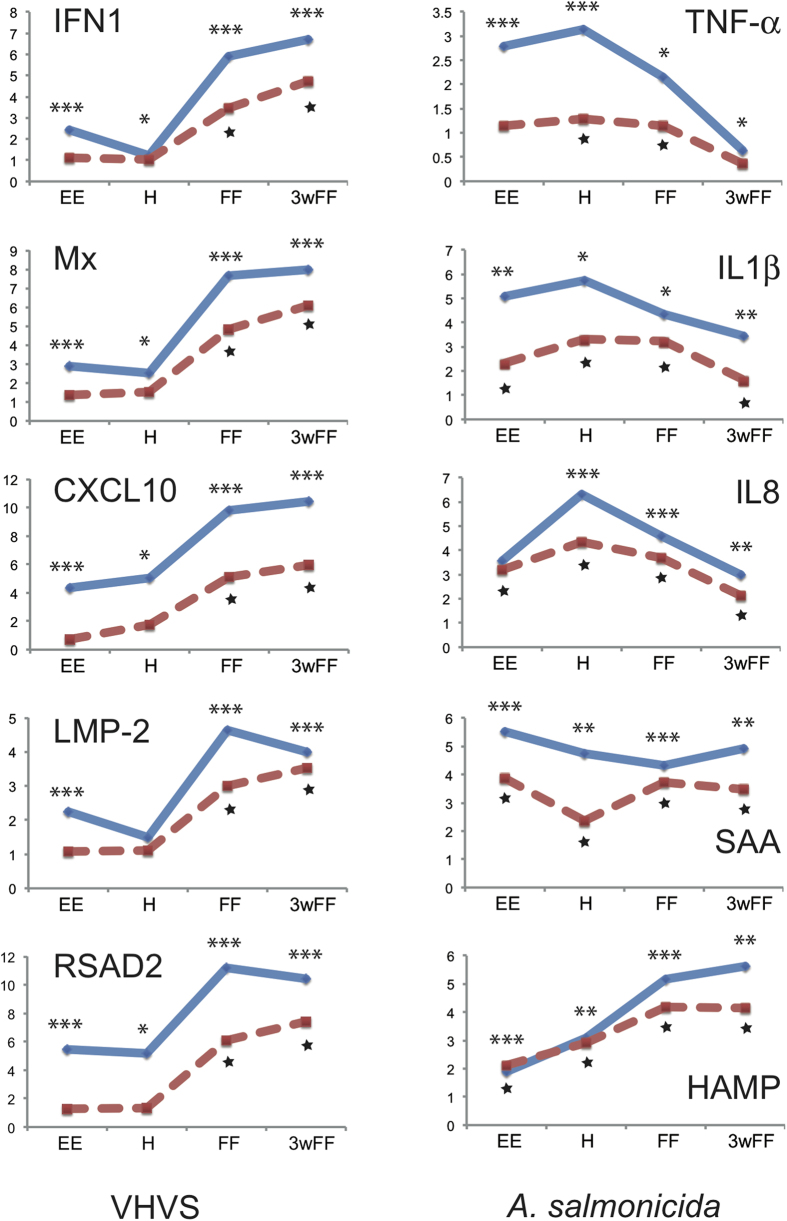
Comparison between transcriptomics responses of selected genes measured by micro-array (red dotted line) and by real time PCR (blue solid line). Data are represented as Log_2_ratios of expression levels (infected/control). The complete qPCR dataset is in Table S3. Significant difference between infected and control fish are denoted by * for qPCR (*p < 5%; **p < 1%; ***p < 0.1%) and by ⋆  for micro arrays. For all ANOVA tests on qPCR results, the assumptions of normality and homoscedasticity were evaluated on model residuals using the Anderson-Darling and Levene’s tests, respectively. When data did not conform to these expectations, transformations were tested, including the Box-Cox and double square root. Data that deviated strongly from normality and homoscedasticity, even after trialling multiple transformations, were analysed using a nonparametric Kruskal-Wallis test. The qPCR data analysis is detailed in Supplementary method.

A panel of 10 genes with various patterns of induction across development were used for qPCR analysis as validation of the microarray results (Fig. 3, Table S3). These experiments confirmed the expression patterns determined by microarray hybridization.

### Functional pathways diverge during development in a pathogen specific fashion

To further characterise the responses we used both gene ontology (biological process) enrichment analysis and KEGG pathway analysis to identify key functional groups that were enriched across each developmental stage. Results from this analysis showed a clear difference between the antiviral and antibacterial transcriptional response (Table S4). The enrichment of biological processes in viral infected fish increased from 5 GO identifiers in EE to 115 in 3wFF (Benjamini corrected *e* value < 5%) whereas the number of GO enriched biological processes in bacterial infected fish was much more consistent over age with 38 in EE and 59 in 3wFF.

#### Responses to VHSV infection: maturation of the IFN response from the FF stage

**Figure 4:**
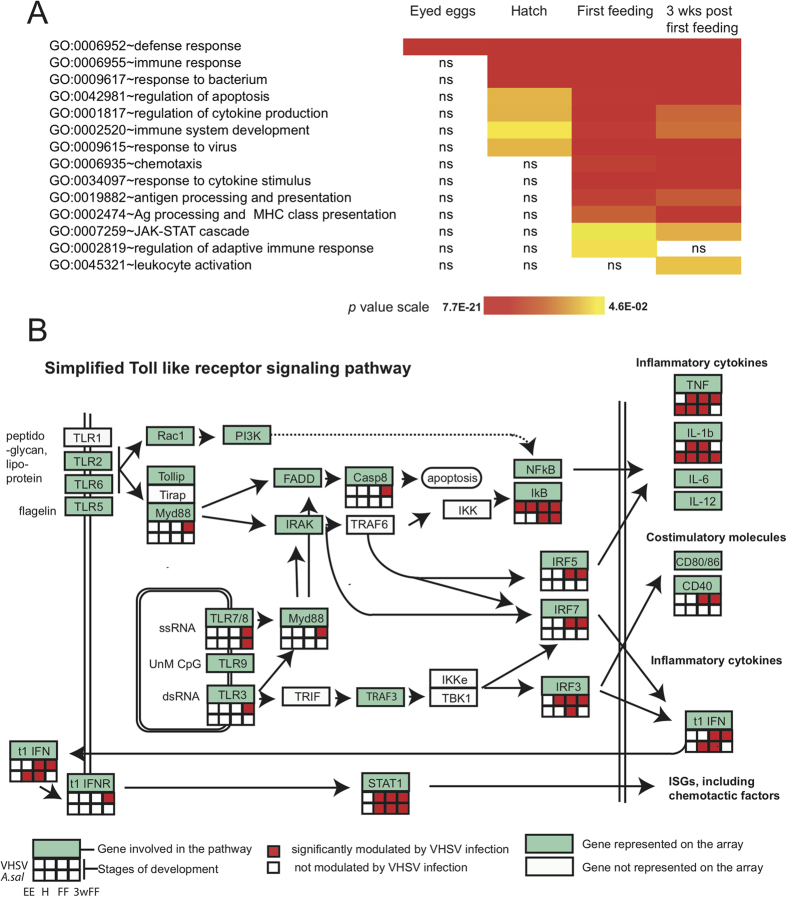
Functional analysis of the transcriptome response to VHSV. (**A**) Enrichment of selected GO terms (BP, GO fat, from DAVID at http://david.abcc.ncifcrf.gov/) at different stages: eyed eggs “EE”; hatching “H”; first feeding “FF” and 3 weeks post first feeding “3wpFF”. Relative significance is depicted as a heatmap of p values (Benjamini correction). “ns” is for non significant enrichment at a given stage. (**B**) Analysis of gene expression responses in the TLR pathway upon VHSV infection. Virus modulated genes (adj. *p *< 0.01; FC > 2 or <0.5) at the four studied developmental stages were mapped on a simplified TLR pathway based on knowledge from mammals. White denotes genes that were not represented on the array platform. Analysis of gene expression in the RLR pathway is represented on figure S1.

For the viral responses, GO terms that were significantly enriched confirmed a major change in transcriptional activity. The magnitude of the enrichment (that is the significance of a GO biological process being over represented in an experimental group) is shown in Fig. 4A. At all stages generic defense response processes are highly enriched in all groups, as seen for “defence response” and “inflammatory response”, with a clear increase in the level of significance with ontological stage. A second set of biological processes with immune genes involved in “antigen presentation” and “lymphocyte proliferation” is significantly enriched only at FF and 3wFF, reflecting the maturation of adaptive immunity. Most interestingly, the terms covering the virus specific response such as “Jak/Stat pathway” and “response to viruses” were processes most significantly enriched at the two later stages, suggesting that the typical virus-induced response is not fully expressed at EE and H stages. Although the smaller number of features used for the analysis at the EE stage can decrease the sensitivity of this approach, these observations suggested that a full interferon response is not expressed early in fry development.

To test this hypothesis, we performed a specific analysis of gene expression in Toll-like receptor and RIG-I signalling pathways. We mapped up- and down-regulated genes on KEGG for TLR and RLR pathways at each developmental stage, using a combination of David^19^, Ingenuity pathway analysis (IPA) and direct analysis of the dataset. As shown on Fig. 4B and Figure S1, most inducible genes of both pathways were not significantly up-regulated at EE, but were at later stages.

A selection of genes up-regulated by the VHSV infection (comprising probes modulated with the 20 highest FC at least for one developmental stage; “top20” genes) is shown in Table 1. For this selection, two main sets of virus induced genes could be distinguished: (1) “top20” genes induced by the virus, and not among the “top20” genes induced by *A. salmonicida* at any stage of development (set 1) and (2) genes that are among the “top20” for both pathogens for at least at one stage (set 2) (Table 1).

When arranged from early to late genes, the expression heatmap reveals significant patterns. Within set 1 (“VHSV specific Top20 genes”) that comprises many well known interferon stimulated genes (ISGs) including viperin, CD9, ISG15 and others, fold changes greatly increase at the later stages. These genes are generally not induced by the bacterial infection, or only at low magnitude in later stages. These observations indicate that a strong, typical type I IFN response is triggered only at FF and 3wFF stages.

In contrast, set 2 (“VHSV and *A. salmonicida* shared Top20 genes”) comprises genes up-regulated at most stages and do not generally show higher fold changes at later stages. These genes are also well-induced by *A. salmonicida* infection at most stages, and are mainly inflammatory and acute phase effectors such as antimicrobial peptides, serum amyloid proteins, and metalloproteinases. A few genes of set 2 (bottom of the panel) show more stage-restricted induction patterns, but these patterns are parallel after viral and bacterial infection which also suggests roles in general inflammation.

Importantly, this was not restricted to the “top20” gene list. Indeed, 85% of genes modulated by the virus only (*ie.* not by the bacteria at any stage), were induced only at 3wFF (46%) or only at FF and 3wFF (39%). Furthermore, among genes modulated by both VHSV and *A. salmonicida*, 70% of genes modulated at three or four stages after viral infection were also modulated at least at three stages after bacterial infection.

Regarding genes down regulated by VHSV infection (Table S1), GO analysis identified “aromatic compound catabolism” and “aromatic amino acid family metabolism” as significant biological processes. It is interesting to note that tryptophan dioxygenase and tryptophan hydroxylase are down-regulated at FF and 3wFF, when the interferon response is well developed. However, while tryptophan catabolism by a related enzyme (indoleamine 2,3-dioxygenase) is a major component of the IFNγ response against Hepatitis B virus^20^, a role of this pathway in defense against RNA viruses remains to be established. Repressed genes also include CD248 (endosialin), a surface glycoprotein of vascular endothelial cells; this likely reflects that vascular endothelial cells are a major target of the virus.

#### Transcriptome changes following A. salmonicida infection

Following the bacterial challenge all stages showed a major transcriptional response to the pathogen. Key biological processes that are enriched include “acute inflammatory response”, “innate immune response” and “humoral immune response” (Figure S2A). These GO identifiers include many key genes well characterised in inflammation such as IL-1β, transcription factors such as CEBP and cell surface lectins. We found that there is little consistent increase in the significance of the enrichment with ontological age and that many of the key genes up-regulated show a similar response at all stages. We did observe that the number and significance of enriched biological processes was reduced at the hatch stage; however, this stage had the highest number of genes altered in response to the infection which appears to impact the relative enrichment value. The major host response to *A. salmonicida* is an antibacterial response (Table 1 and Table S2) with cathelicidin and hepcidin, two well characterised antimicrobial peptides, being highly increased at all stages. In parallel it is of interest that the acute inflammatory response was highly enriched, even at EE, suggesting that this response can be highly up-regulated in the embryo. At the EE stage, genes driving inflammation such as IL-1β, IL-8 and TNF were also up-regulated. In addition genes involved in signalling such as the transcription factors JunB, C/EBP and MARCKS- like protein kinase suggest that there is a comprehensive bacterial response from an early developmental stage. This could be clearly seen when a KEGG pathway map was generated for complement activation (Figure S2B), showing that the components of this innate immune process are highly activated from the earliest stages.

To counteract the inflammatory response, transcripts encoding anti-inflammatory factors such as SOCS3 were increased in expression at all stages, however IL-10 was not increased in these fish. The GO identifiers related to cellular homeostasis are enriched at the later stages of development and include transferrin, ferritin and other serum proteins such as serum amyloid A (Figure S2; Table 1). Although the total number of genes responding to the bacterial pathogen increases with age, many of these can be categorised within the general immune function. Whilst the overall functional groups are similar over development there are some interesting genes that are not found expressed during the earlier stages, and include some cell surface receptors such as the pathogen recognition receptors, C-type lectins and mannose binding lectins, and the TRL5 receptor (which is not increased in expression in either E or H stages). These PRRs act through intermediate adaptor proteins including MYD88 and TRAF6 (both present on the microarray but not altered) and finally responsive genes via the NFκB transcription factor. The NFκB inhibitor protein that regulates NFκB nuclear import is not expressed in the EE stage but does become expressed at all later stages. Additionally during the egg stage the GO terms for iron and cellular ion homeostasis are not enriched, suggesting that overall cellular homeostasis matures as the fish develops.

Among the list of the “top20” genes up-regulated by *A. salmonicida*, and not among the “top20” genes induced by VHSV at any stage of development (Table 1*, set 3, “A salmonicida specific Top20 genes”)*, two patterns could be distinguished: (1) genes that are induced only at early stages (EE and H) by bacterial infection (FOS, MIER1, PNP); they are generally not induced by VHSV and appear as specific markers of the “early” response to bacterial infection, and (2) the other genes of set 3 which are induced at the later stages (FF and 3wFF) by *A. salmonicida*; these genes are often also up-regulated by VHSV at these stages although to a lower magnitude. This pattern is consistent with distinct transcriptional responses to the *A. salmonicida* infection at early and late stages.

Although the majority of genes are increased in expression following the *Aeromonas* infection there are a number of genes that are consistently decreased in expression in all the early life stages. The collagens are the most pronounced genes with col1, 2, 4, 11 and 12 all being reduced in expression. These collagen genes are found in the GO analysis under “extracellular matrix organization” and “collagen metabolic process”. A second group of genes consistently down regulated encode structural proteins and are related to the GO term “muscle system process”. They include a variety of myosin heavy chain genes in several stages and myomesin, a protein key to myofibril development in striated muscle, which is decreased at all stages following bacterial infection. Together these results show that muscle growth and structural organization is being inhibited, most likely due to energy (incl. cellular energy) being directed towards immune function.

### The repertoire of genes co-modulated by VHSV and *A. salmonicida* evolves during development

To investigate further the extent to which transcriptional responses to viral and bacterial challenge overlap during early trout development, we determined the numbers of genes co-modulated at each stage of development (Figure S3). The proportion of genes up- or down-regulated by both pathogens represented 10 to 30% of the total number of responsive genes at the studied stages (Figure S3A). In fact, the large majority of the genes modulated by both infections was expressed at FF and/or 3wFF only (253 out of 326), and only 16 genes were common to the intersect at all stages (Figure S3B). Overall, these observations indicate that genes common to the transcriptome response to VHSV and *A. salmonicida* do not represent a large generic set of permanent inflammatory markers inducible from an early stage. On the contrary, this subset of dual responsive genes greatly expands at later stages.

Thus, two main sets of co-modulated genes can be distinguished:

(1) Forty-two genes that are co-induced by viral and bacterial infection in at least three stages (Table S5A). These genes generally follow similar induction profiles across developmental stages for the viral and the bacterial infection. The main functional subsets to which these genes belong are antimicrobial peptides; acute phase proteins like serum amyloid A (SAA), complement and the metalloprotease MMP9; several lectins and chemokines also fall into this gene set. Of interest the genes with the highest fold changes are observed for antibacterial peptides such as cathelicidin and hepcidin, and SAA.

(2) A larger set of genes (247 genes) are co-modulated in viral and bacterial infection only at FF and/or 3wFF stages (Table S5B). This set comprises a number of well characterised ISGs including Mx, viperin, IRF1 and CD9 amongst others that are strongly up-regulated by the virus, while fold changes after bacterial infection are much lower and reflect the reduced magnitude of the IFN response triggered in this context. Interestingly, a few members of a large fish specific TRIM subset discovered as VHSV and IFN induced transcripts are also found within this gene set. The other functional category relies on the complement pathway, as C3, C4B, C6, C7, C9 and MASP were also induced by both infections. These genes are generally induced at comparable, moderate rates by VHSV and *A. salmonicida*. Several lectins also participate in both responses, reflecting their role in general inflammation.

The remaining genes are induced with moderate fold changes (2 to 3), and although most are not directly associated with immune function, demonstrate that many other biological processes alter in the animals during the response to pathogens. They may be up-regulated as a side effect of the inflammatory response, or be involved in metabolic changes related to energy reallocation and lipid metabolism.

Overall, our data identify a gene subset modulated at all developmental stages by both pathogens in which antibacterial peptides appear as key protective factors, and a gene subset induced by VHSV and *A. salmonicida* only at later stages, which comprises ISGs that are highly up-regulated by the virus, and somewhat induced by the bacterial infection.

## Discussion

Teleost eggs and fry come into contact with many pathogens as their immune system matures and whilst their early defence mechanisms are mainly restricted to innate immunity. However such early stages of development are not particularly vulnerable to infections, and we could not infect trout eggs by bath challenge in concentrated viral or bacterial suspensions. In fact, the egg envelope is very effective at preventing pathogen entry, and hatching and first feeding therefore constitute key transitional stages with respect to the interactions of the developing fish with the pathogen world.

Innate immune pathways triggered by viruses and extracellular bacteria are generally considered to be distinct but their role in the defence of the developing fish remains poorly characterized. As zebrafish larvae have been used to set up models for human infectious diseases^21^,^22^, responses against viruses and bacteria have been studied separately at the transcriptome level during development in this species^23^,^24^,^25^,^26^,^27^. These studies were generally performed for a given pathogen administered between 30 and 72 hpf, larvae being sampled 24–72 hpi, *ie.* just around the time of mouth and gut opening/first feeding or before^23^,^24^. Our study provides a diachronic comparative perspective on responses induced by a virus and a bacterium in a salmonid. Since rainbow trout development is slow compared to model species like zebrafish, with a clear temporal separation of stages, it provides an excellent model for such an approach. Importantly, salmonids have very different developmental dynamics compared to zebrafish, with larger energy resources allocated to the egg.

Our data show that the development of virus- and bacteria- induced responses follow different dynamics, highlighting the importance of studying various life stages. The antiviral response shows a progressive development, with a few typical ISGs induced at EE and H stages, and a typical IFN based response at FF, which expands further at 3wFF. Importantly, the master antiviral cytokines, type I IFNs, are not induced at the early stages, raising the issue of the differentiation of IFN- and ISG- producing cells.

The trout data presented here regarding the response to VHSV suggest that IFN is not induced in all cell types and significant transcriptional response is observed between H and FF stages. Later expansion of specialized cells involved in adaptive immunity between FF and 3wFF, which is reflected in expression of genes involved in antigen presentation, likely explains the increase of the ISG repertoire expressed at the most mature stage analyzed. Accordingly, previous studies on response to CHIKV infection, which induced a very strong IFN response in zebrafish larvae (around 5dpf) underlined the key role of neutrophils in IFN production^24^. Furthermore, the liver, gut and blood vessels appeared to be the main sites of ISG expression after CHIKV or IHNV^24^.

In sharp contrast, the response to *A. salmonicida* is already considerable at the EE stage. However, the transcriptome response goes through large-scale and unexpected transitions across the four analyzed developmental stages with about half of the genes induced at EE and H being stage specific. Interestingly, very few of the “top20” induced genes at EE were specific to this stage; thus, genes induced by *A. salmonicida* only at the EE stage are up-regulated (moderately) either in EE stage-specific cells, or by cells that become unresponsive at later stages. In fact, some of the genes that were induced only at the EE stage may be members of gene families in which distinct members with redundant functions are induced at different stages. In this respect, our data provide a resource for understanding natural resistance at early stages and guiding selection.

While specialized responses to viral and bacterial infections trigger different pathways and cell types/tissues, some mechanisms were triggered by both types of pathogen. It is well known that proinflammatory cytokines such as IL-1β and TNFα, which are involved in fever in homeotherms, are induced by many different infectious pathogens. In fact, the actual impact of inflammation on the infecting pathogen and on the well-being/survival of the host is a delicate equilibrium. In our data, two main pathways might be of special importance for the effective defence against both types of pathogens: the complement cascade and the IFN pathway itself.

Our data not only represent the first diachronic comparison between fish responses against a virus and an extracellular bacterium, they also provide an excellent platform to compare the maturation of the pathogen sensor-induced responses between fish and mammals. In humans, four main phases have been proposed recently in the development of the immune system^17^. A first phase before birth with low immune responses and expression of anti-inflammatory cytokines such as IL-10. A second phase around birth, when the new-born blood cells produce large amounts of IL-6 and IL-23 after TLR stimulation, supporting TH17 cell differentiation while the type I IFN responses are very low. A third phase when type I IFN responses reach the level of the adult a few weeks after birth, and lastly the fourth period when the proinflammatory and Th1 cytokine responses including expression of IL-1β and TNFα develop progressively over the first years of human life. Our data offer a first opportunity to establish a parallel between these distinct phases in humans and fish.

This immunosuppressed pattern of the first phase is consistent with the quasi sterile environment of the foetus *in utero*, and with a need for mutual tolerance between mother and foetus. In teleosts, the EE stage is also well protected by its envelope, but does not need to develop any tolerance to mother. However, vertical transmission might occur for fish bacterial pathogens, when capacity to respond is required. Our data suggest that the antibacterial response is significant, and we did not detect any induction of IL-10 or other regulatory cytokine; the IL-10R is in fact down-regulated in eggs injected with *A. salmonicida*. The hatching of fish eggs is hardly equivalent to birth in humans and there are certainly no mature T cells at this stage. However, the type I IFN response is still low, while orthologues of several interleukins or interleukin receptors that may be involved in Th17 differentiation in mammals are induced at this stage by *A. salmonicida* (IL-11 and IL-21R)^28^ or by the viral infection (IL-17RA). In trout, the amplitude of the type I IFN response and the diversity of expressed ISGs is greatly increased at the FF compared to EE and H stages, and appears to be quasi complete by 3wFF. This is in good agreement with the high susceptibility of young fry to viruses, including viruses that are specific to other species at the adult stage. For example, we observed that zebrafish embryos are efficiently killed by a IHNV strain adapted to high temperature, while older fish are much more resistant to the same virus with mortality rate generally <50%^29^. Important differences are also observed with the human fourth phase as the proinflammatory and Th1 cytokine response including IL-1β and TNFα develop progressively over the first years of the human life, but IL-1β induction by the virus or *A. salmonicida* did not clearly increase in trout from EE to 3wpFF. In contrast, the IL-2 receptor (CD132) was not modified during the EE stage but was increased at later stages, which might suggest a greater level of maturation of the Th1 cell lineage.

## Conclusions

We find that during the ontological development of teleosts the responses take different trajectories depending on whether the immune stimulus is viral or bacterial. Our data allow us to draw a parallel between these changes to the mammalian “phases” of immune ontology.

## Methods

### Ethics statement

All animals were handled in strict accordance with good animal practice as defined by the European Union guidelines for the handling of laboratory animals^30^ and by the Regional Paris South Ethics committee, and all animal work was approved by the Direction of the Veterinary Services of Versailles (authorization number 78-28).

### Fish infections

A rainbow trout double haploid clone, named B57^31^, was used for immune challenges as Eyed eggs (EE) at 200 DD, or fry at the time of hatching (H) 370 DD, first feeding (FF) 560DD, and 3 weeks post-first feeding (3wFF) 770DD. Three groups of 40 individuals were bathed in 1l of water (control), water with VHSV (10^5^ pfu per ml), or with *A. salmonicida* (10^6^ pfu per ml) for 24 h, and samples collected at 3 and 6 days post infection. For the injection challenge, 20 individuals of each stage were injected with 1 μl of PBS as uninfected treatment control, 1 μl of VHSV (10^8^ pfu per ml), or 1 μl of *A. salmonicida* (10^6^ pfu per ml), and collected 3 days post infection. At the EE stage there was 30% and 50% mortality within 24 h in the VHSV and *A. salmonicida* groups respectively, suggesting mechanical damage to these eggs. No significant mortality due to the injection was observed in other groups. The experiments were performed at 10°C.

### Microarray platform, hybridizations and analysis

The microarray platform was custom designed and derived from rainbow trout EST tentative contigs (TC) from Rainbow Trout Gene Index Release 7.0 (July 2008). 40,125 oligos from this source were the basis of the array, and were supplemented with further immune related transcripts from more recent releases to NCBI designed using the Agilent e array oligo design software (1,287 oligos). A further 1,417 Agilent in house control features were included. Full details of the array platform are available at EBI array express ( http://www.ebi.ac.uk/arrayexpress/ : “Trout_imm_v1”_(Agilent array design 028918) with platform accession A-MEXP-2315. The hybridizations were performed as described in Supplementary methods, and the data have been deposited at EBI array express under accession number E-MTAB-3401. Primers used in this study are shown in Table S5, and the QPCR methods are detailed in supplementary data.

## Additional Information

**How to cite this article**: Castro, R. *et al.* Disparate developmental patterns of immune responses to bacterial and viral infections in fish. *Sci. Rep.* **5**, 15458; doi: 10.1038/srep15458 (2015).

## Supplementary Material

Supplementary Information

Supplementary Information

Supplementary Information

Supplementary Information

## Figures and Tables

**Table 1:**
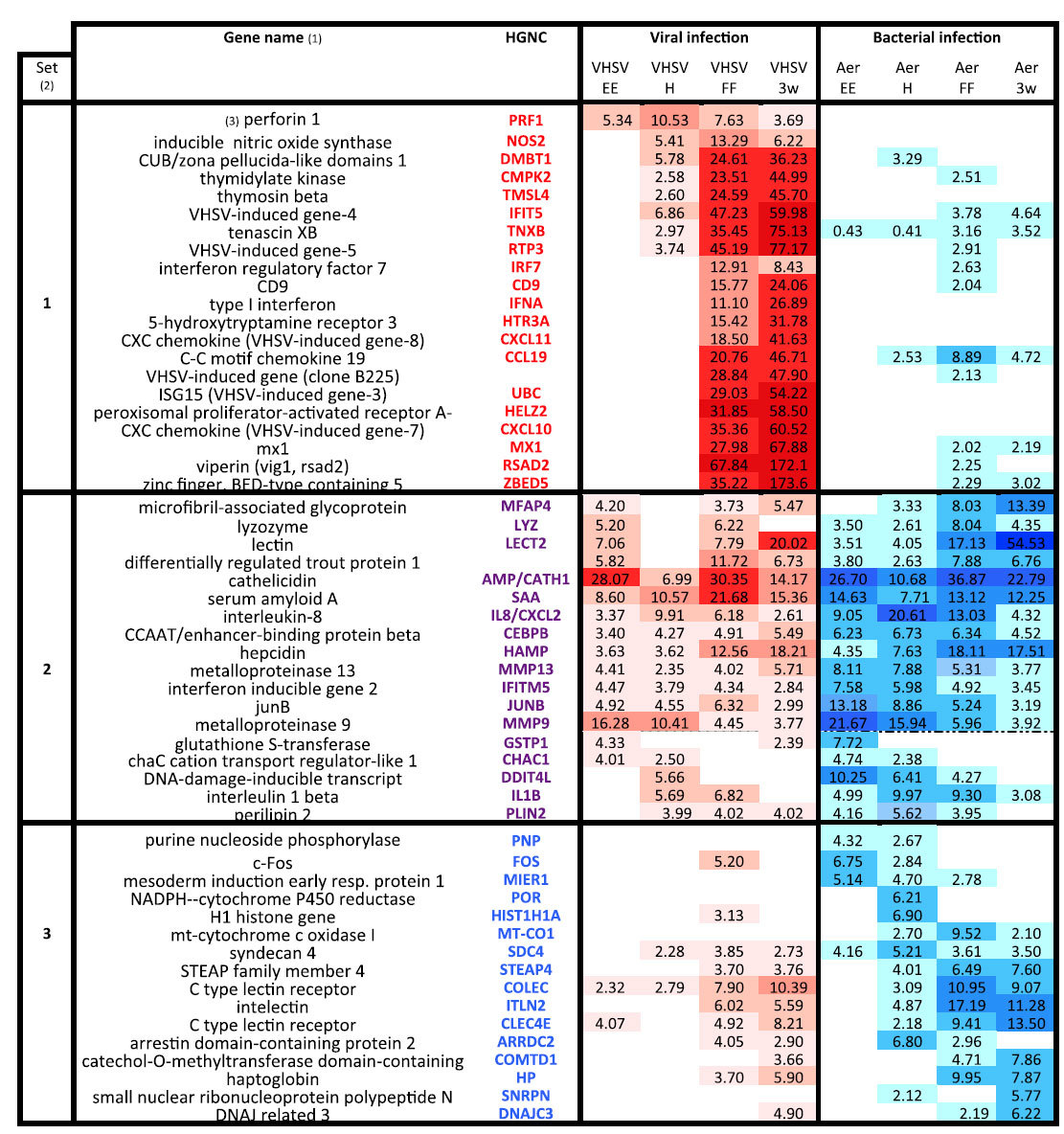
Top up-regulated genes after VHSV and *A. salmonicida* infection show pathogen-specific expression patterns.

(1) Lists of the top up-regulated genes after viral or bacterial infection were produced by collecting probes induced with the 20 highest fold change (»top20») values at the different developmental stages. The list was manually curated to keep distinct genes with available functional annotation.

(2) HGNC of genes only present in the top up-regulated list after viral infection are in red (set1); the intersect of the lists of the top up-regulated genes by viral and by bacterial infection has HGNC in purple (set2) HGNC of genes onlypresent in top up-regulated list after bacterial infection are in blue (set3).

(3) The order of probes was optimized to depict the dynamics of the response across development, from probes induced only at early stages to probes induced at all stages, and finally to probes induced only at later stages; the reference being the viral infection (set1), the bacterial infection (set3) or both (set2), according to the distribution of the “top20” genes. FC values after viral and bacterial infection are highlighted as heatmaps (red after VHSV, blue after A. salmonicida) that reveal expression patterns emerging when probes are classified in this way.
