# Corrigendum: Disparate developmental patterns of immune responses to bacterial and viral infections in fish

**DOI:** 10.1038/srep18524

**Published:** 2016-01-27

**Authors:** Rosario Castro, Luc Jouneau, Luca Tacchi, Daniel J. Macqueen, Abdullah Alzaid, Christopher J. Secombes, Samuel A. M. Martin, Pierre Boudinot

Scientific Reports 5: Article number: 1545810.1038/srep15458; published online 10212015; updated: 01272016

There is an error in the Materials and Methods section of this Article.

“A rainbow trout double haploid clone, named B57^31^”

should read:

“Rainbow trout from the INRA ‘synthetic’ strain^31^”

