# Supplementary material for: Disparate developmental patterns of immune responses to bacterial and viral infections in fish
Source: Sci Rep. 2015 Oct 21;5:15458. doi: 10.1038/srep15458 (PMC4614352; doi:10.1038/srep15458)
Supplement: Supplementary Information [file srep15458-s1.doc]

**Disparate developmental patterns of Immune responses to bacterial and viral infections in fish**

**Supplementary material**

Rosario Castro^1^, Luc Jouneau^1^, Luca Tacchi^2^, Daniel J Macqueen^2^, Abdullah Alzaid^2^,

Christopher J. Secombes^2^, Samuel A. M. Martin^2#*^, Pierre Boudinot^1#*^

^1^Virologie et Immunologie Moléculaires, INRA, Jouy-en-Josas, France

^2^Institute of Biological and Environmental Sciences, University of Aberdeen, Aberdeen, UK, AB24 2TZ

* Corresponding authors

^#^ These authors contributed equally to the work.

**Table of content**

**Supplementary figure 1.** Analysis of gene expression responses in the RIG-I pathway upon VHSV infection.

**Supplementary figure 2.** Functional analysis of the transcriptome response to *A. salmonicida*.

**Supplementary figure 3**. Analysis of common transcriptome response to VHSV and *A. salmonicida.*

**Supplementary table 1.** Complete description of features deposited on the micro array significantly up- or down-regulated by VHSV infection (adj. *p* <0.01, FC>2 or <0.5) (separate excel file)

**Supplementary table 2.** Complete description of features deposited on the micro array significantly up- or down-regulated by *A. salmonicida* infection (adj. *p*.<0.01, FC>2 or <0.5) (separate excel file)

**Supplementary table 3.** QPCR validation of micro array results

**Supplementary table 4.** GO analysis of genes regulated by VHSV and *A.salmonicida* infection. (separate excel file)

**Supplementary table 5.** Genes induced by VHSV and *A. salmonicida* at one stage or more.

**Supplementary table 6.** Primers used in this study.

**Supplementary method.** Additional information regarding micro array hybridization and microrarray data analysis.


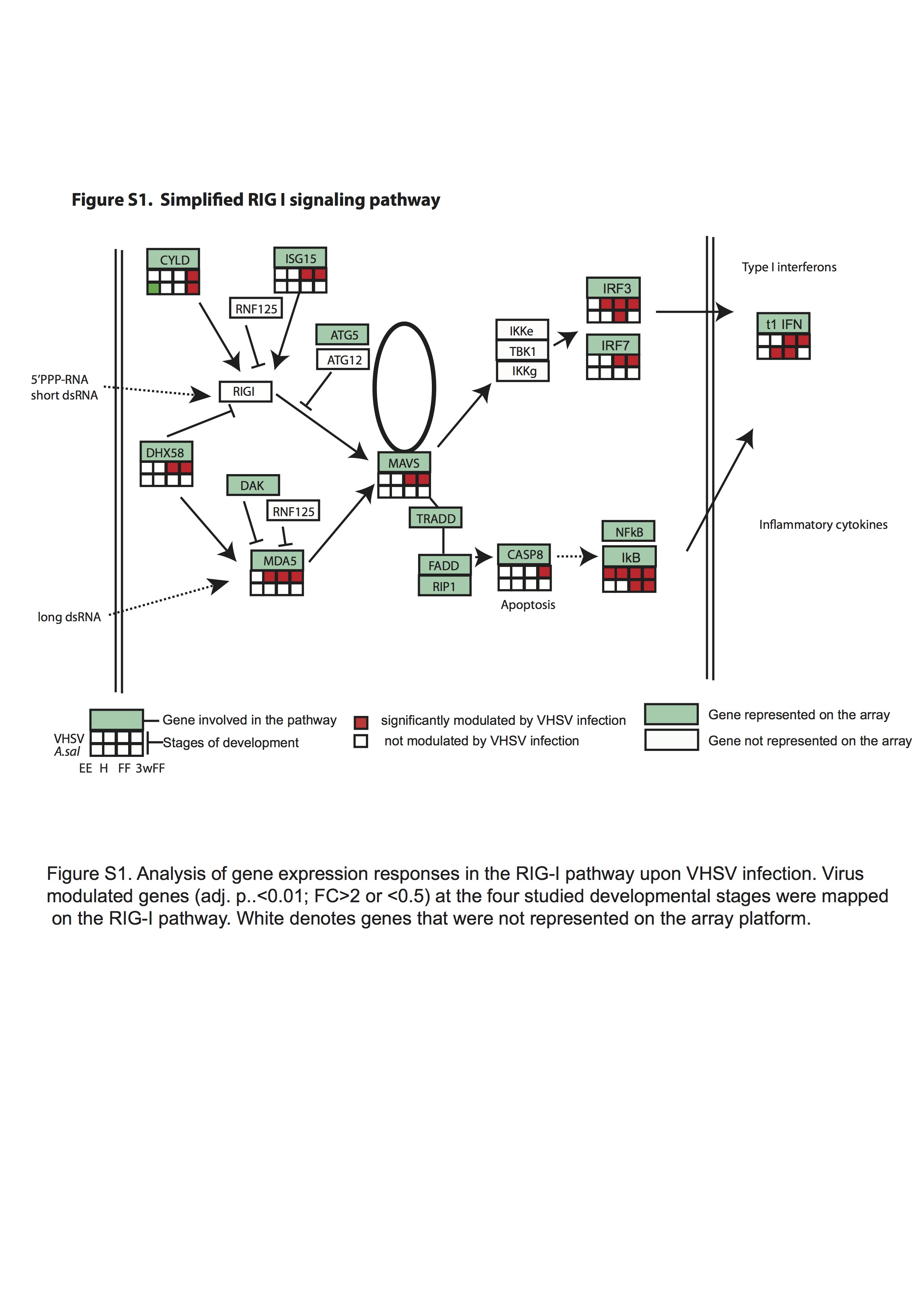


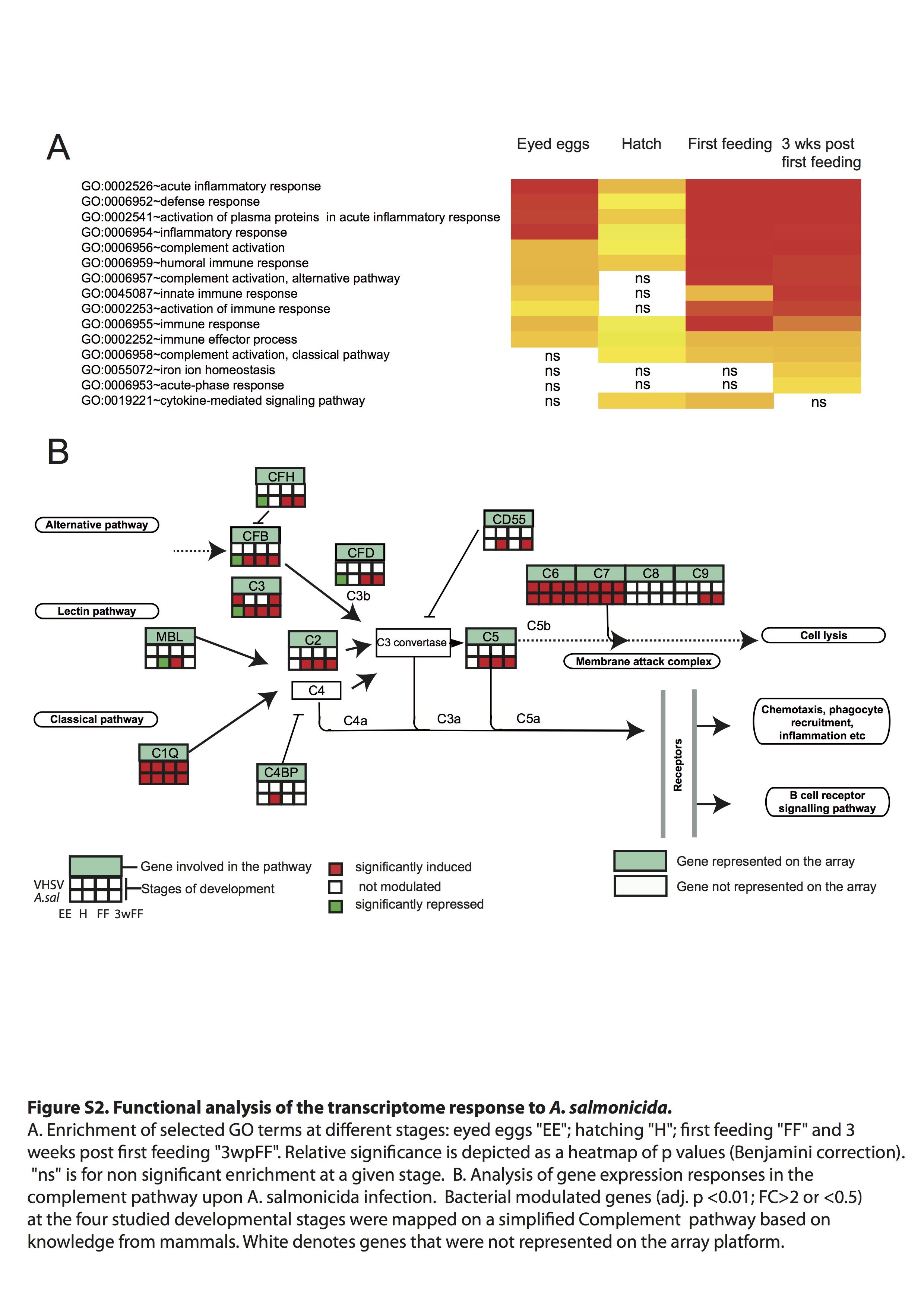


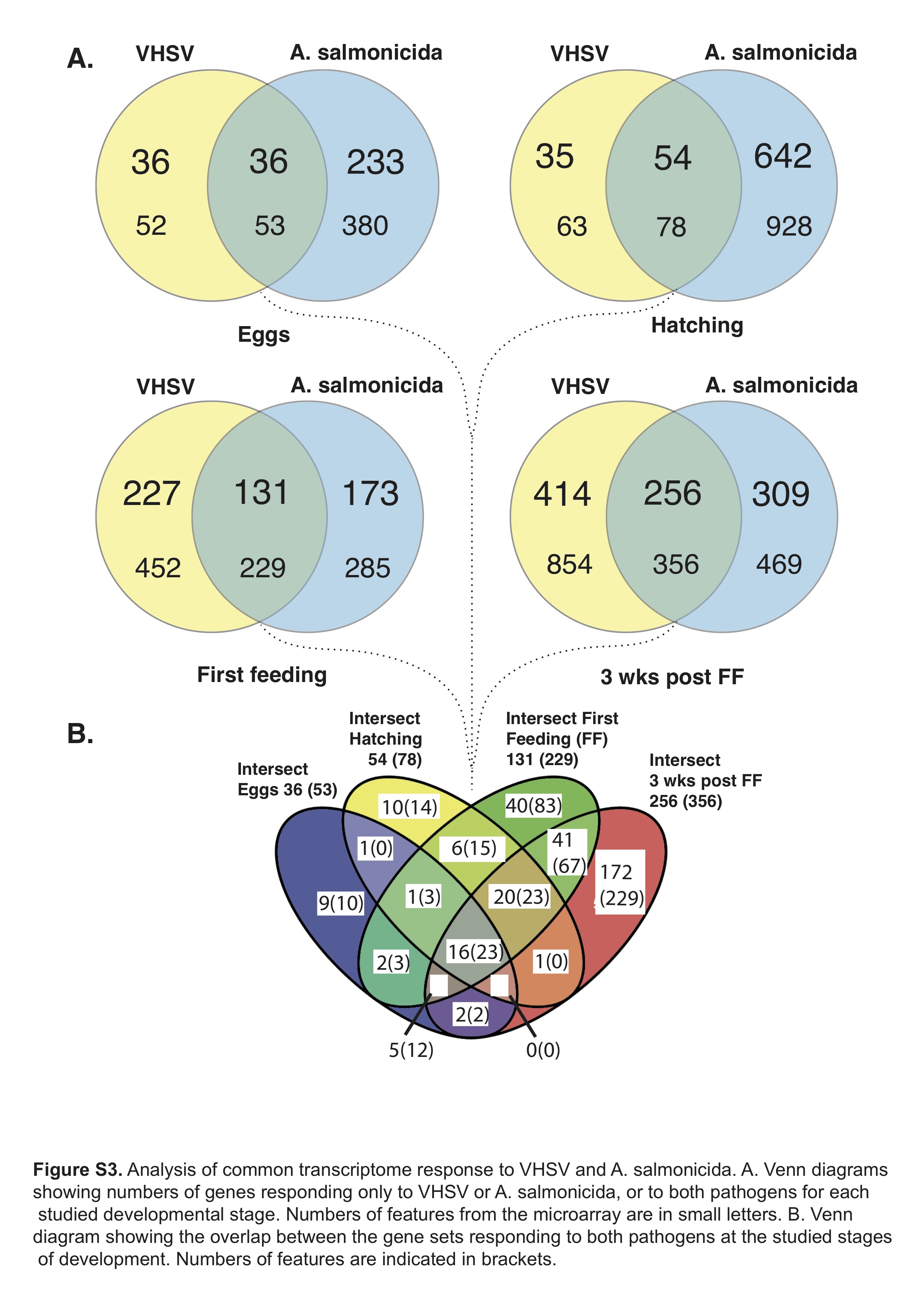


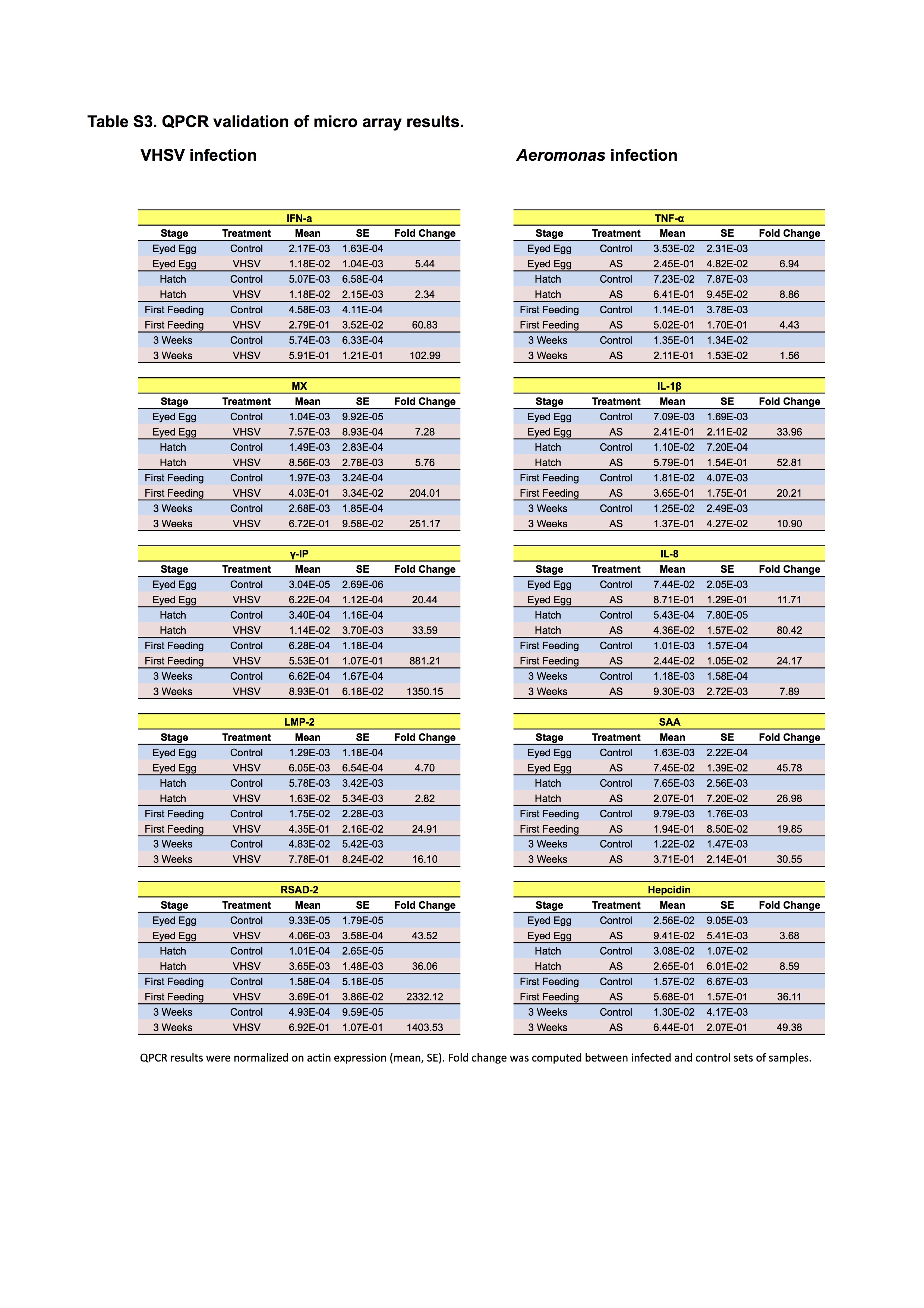


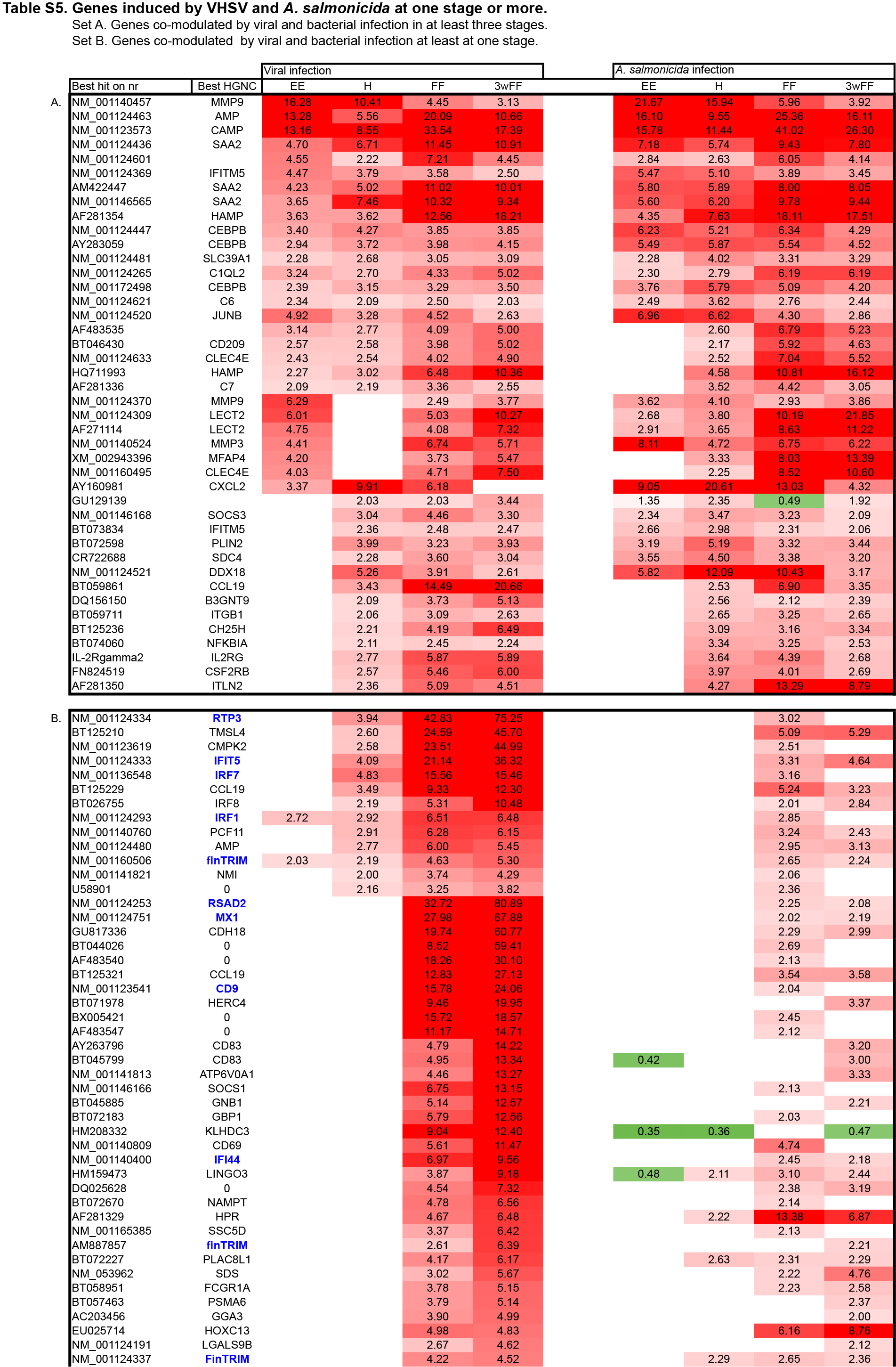


**
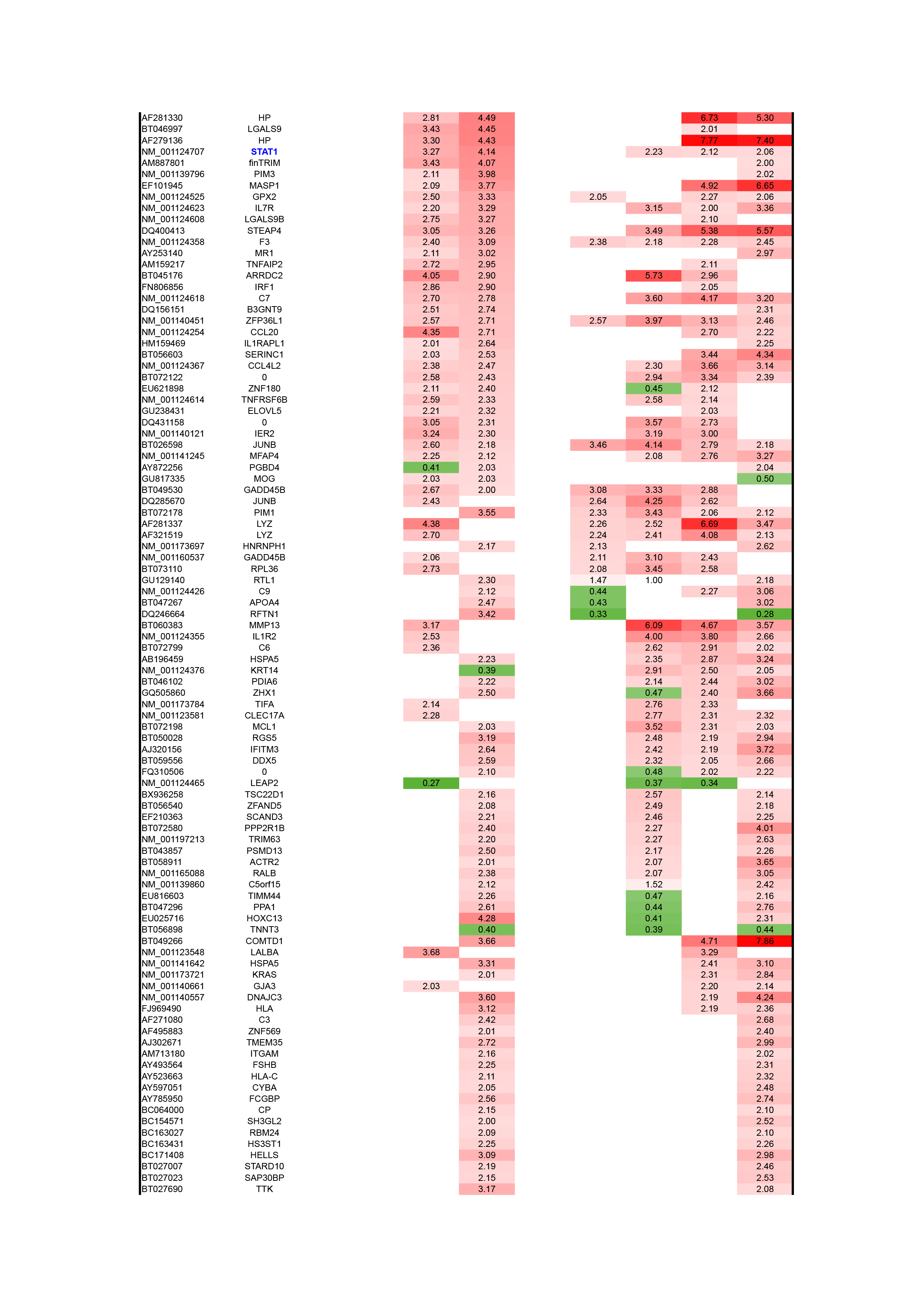
**

**
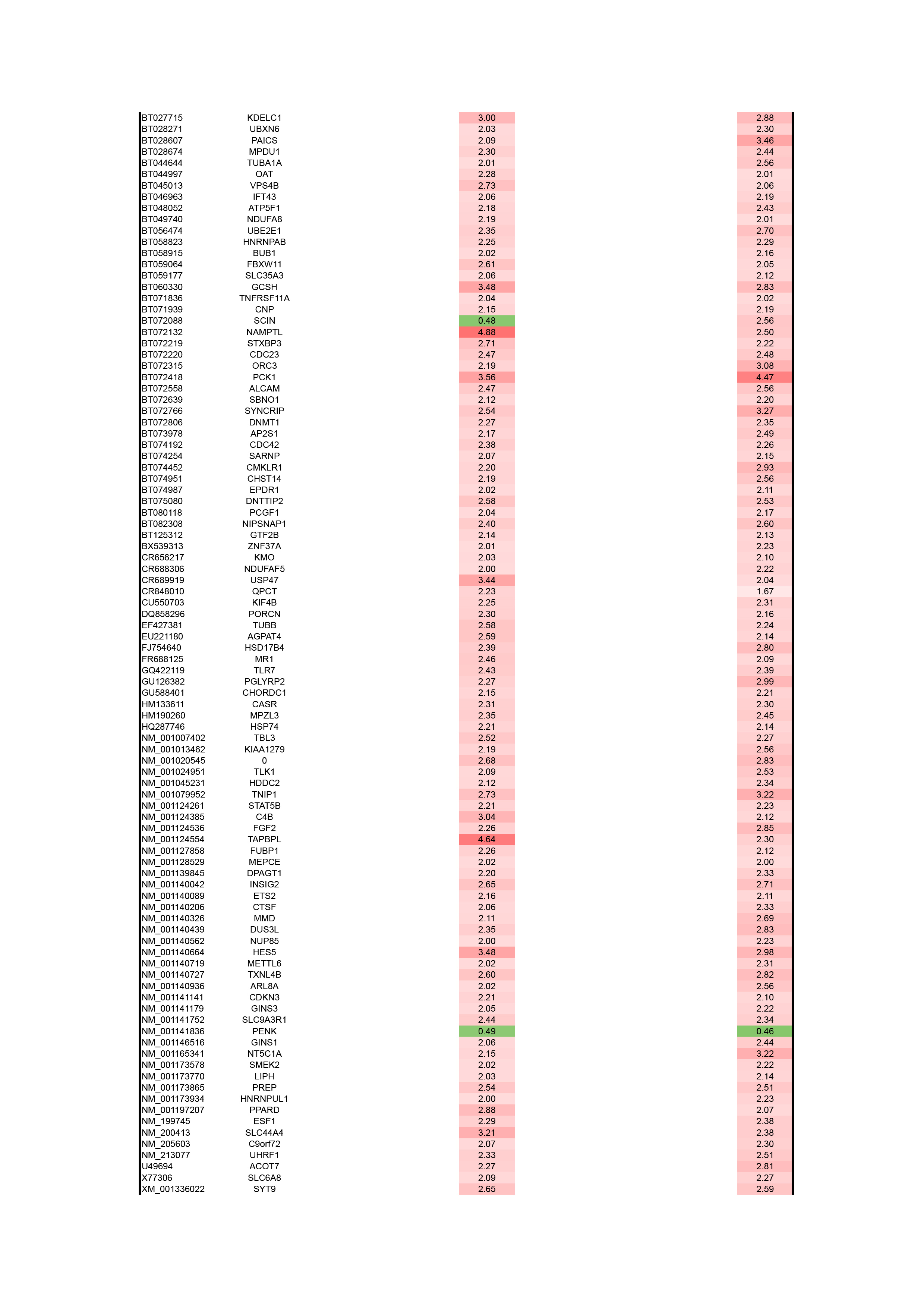
**

**
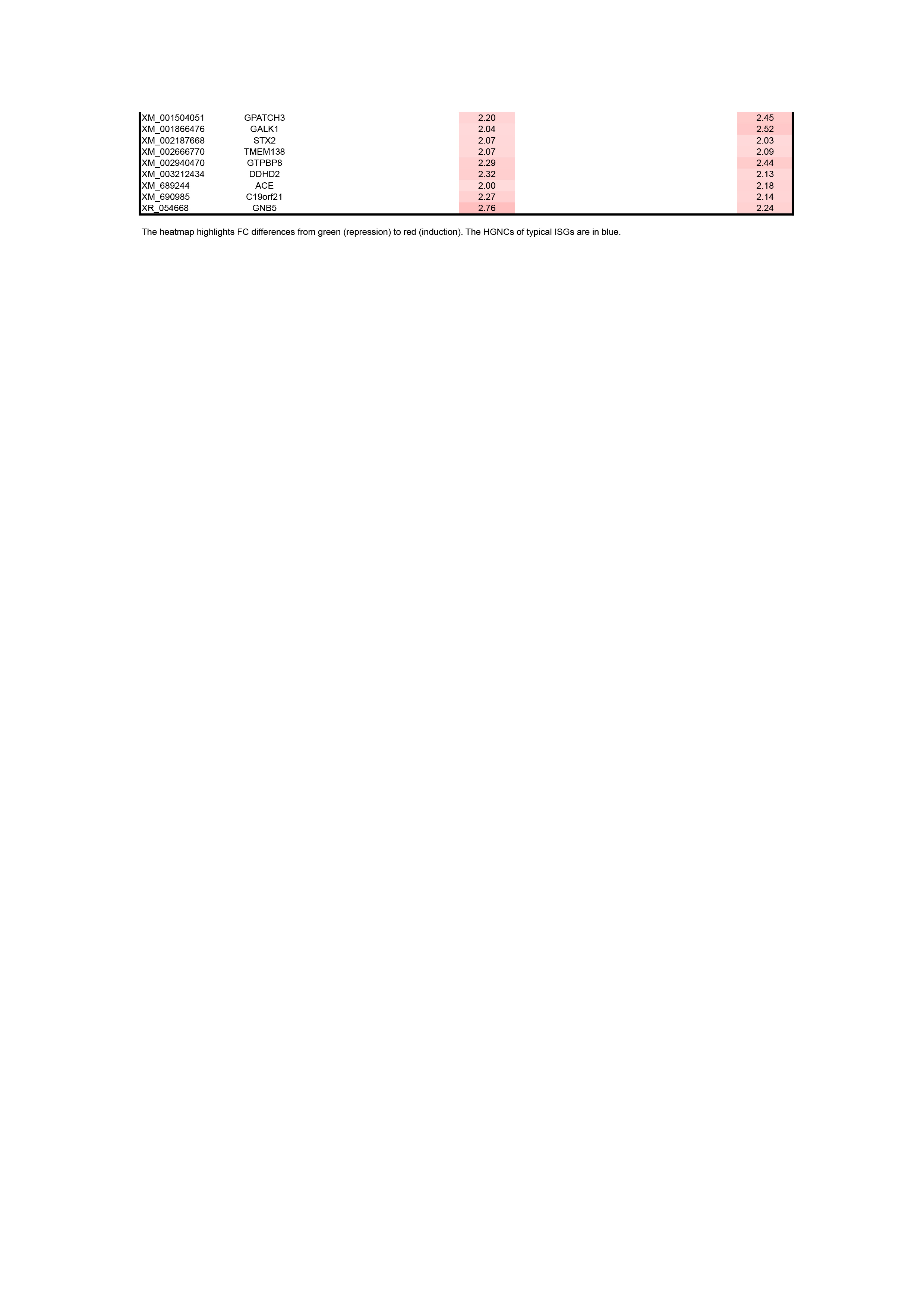
**

**Table S6. Primers used for real time PCR**

| **Gene** | **Sense Primer (5´-3´)** | **Antisense Primer (5´-3´)** | **Product Size (bp)** | **Accession Number/Ref** |
| --- | --- | --- | --- | --- |
|  |  |  |  |  |
| TNF-α | AGAGGTTGGCTATGGAGGCTG | CCGTCATCCTTTCTCCACTGC | 266 | NM001124357 |
| IL-1β | GCCAACCTCATCATCGCCA | GCCGACTCCAACTCCAACAC | 140 | AJ223954 |
| IL-8 | ATGAGCATCAGAATGTCAGCCAG | TGACATCCAGACAAATCTCCTGAC | 241 | AY160987 |
| SAA | TATGATGCTGCCAGGAGAGGAC | CGTCCCCAGTGGTTAGCCTT | 137 | AM422446 |
| Hepcidin | AGGAGGTTGGAAGCATTGACAG | GTGGCTCTGACGCTTGAACCT | 101 | HQ711993 |
| IFN-a | GTGTGTCATTGCTGTGACTGGA | TTTGTGATATCTCCTCCCATCTG | 95 | AJ580911 |
| MX | CGTCCCAGACCTCACACTCATC | TGCCATCTTCAAAGCCTCTGTG | 187 | OMU30253 |
| γ-IP | GACATCAACGGTCCTCATCAGC | CCGTTCCTCAGAGTGACAATGAT | 201 | AJ417078 |
| LMP2 | GTGCTGGGCTCTGACTCTCG | TCTCCAACCTCAATGCTGTGC | 179 | AF112117 |
| Viperin | GGCAACTCCAAGCAGTGTCAA | GTCGTGTATGAAAGGCTCTCCG | 187 | AF076620 |
| RpL4 | CCTTCAGAAACATCCCTGGTATCAC | GGGCAGATTGTAGTCTACCTTGAGAG | 182 | BT057966, [1] |
| EF-1α | CAAGGATATCCGTCGTGGCA | ACAGCAAAACGACCAAGAGG | 327 | AF321836, [2] |
| **Pathogen** |  |  |  |  |
| *A .salm.* | TAAAGCACTGTCTGTTACC | GCTACTTCACCCTGATTGG | 144 | M64655, [3] |
| VHSV-N | CCTGGTGAACAGGTGTCCTT | TTCATAGAGGGGGTTTGCAC | 125 | [4] |

References:

[1] Macqueen, D.J., Kristjánsson, B.K. & Johnston, I.A. 2010 Salmonid genomes have a remarkably expanded akirin family, coexpressed with genes from conserved pathways governing skeletal muscle growth and catabolism. *Physiological Genomics* **42**, 134-148. (doi:10.1152/physiolgenomics.00045.2010).

[2] Tacchi, L., Bickerdike, R., Secombes, C.J., Pooley, N.J., Urquhart, K.L., Collet, B. & Martin, S.A.M. 2010 Ubiquitin E3 ligase atrogin-1 (Fbox-32) in Atlantic salmon (Salmo salar): Sequence analysis, genomic structure and modulation of expression. *Comparative Biochemistry and Physiology, Part B* **157**, 364-337. (doi:10.1016/j.cbpb.2010.08.004).

[3] Keeling, S.E., Brosnahan, C.L., Johnston, C., Wallis, R., Gudkovs, N. & McDonald, W.L. 2013 Development and validation of a real-time PCR assay for the detection of Aeromonas salmonicida. *Journal of fish diseases* **36**, 495-503. (doi:10.1111/jfd.12014).

[4] Verrier, E.R., Langevin, C., Tohry, C., Houel, A., Ducrocq, V., Benmansour, A., Quillet, E. & Boudinot, P. 2012 Genetic resistance to rhabdovirus infection in teleost fish is paralleled to the derived cell resistance status. *PloS one* **7**, e33935. (doi:10.1371/journal.pone.0033935).

**Supplementary method: information regarding Microarray hybridization and Micorarray data analysis.**

*Microarray hybridization*

For microarray analysis, 4 pools of RNA were made from VHSV or *A. salmonicida* challenged individuals and from control individuals, each pool comprising an equal quantity of RNA from three different individuals chosen randomly from each experimental group. A common reference design was used, which comprised an equimolar mix of RNA extracted from all individual fish. Each experimental sample was hybridized against this reference sample in a 2-colour experiment. To generate fluorescently labelled RNA for hybridizations, a MessageAMP^TM^ aRNA Amplification Kit (Ambion) was used for initial amplification of mRNA. The hybridizations were performed in a Microarray Hybridization Oven (Agilent) overnight (18 h) at 65^o^C, and slides were scanned using a GenePix personal 4100A Scanner (Axon Instruments) at a resolution of 5 µm. Images were extracted and initial analysis was performed by Feature extraction v9.5.3 (Agilent). The microarray data has been deposited at EBI array express under accession number E-MTAB-3401.

*Analysis of micro-array data*

Normalization of micro-array signals was first performed using the NormalizeWithinArrays function of the limma R package (with no background correction and duplicated probes averaged). All experiments with a given pathogen (either *A. salmonicida* or VHSV) and those from control samples were then used to build a linear model taking into account all developmental stages used in the experiments (using the function lmFit of the limma package). Differential expression analysis between infected samples and control samples was performed for each developmental stage. P values were corrected for multiple testing using the Benjamini-Hochberg procedure. We considered Adjusted *p* values lower than 1% with an absolute fold change of expression greater than 2 were considered for subsequent analyses. When necessary, the average of signals for probes linked to the same Ensembl gene was considered.

Regarding annotation, long oligo sequences (from the TIGR Gene indices database) or sequences from proprietary clones deposited on the array were used as queries for blastx analysis to the human PepAll Ensembl database. Best blast hits were considered for annotation when the E value was lower than 10^-3^; description, associated gene name (HGNC) and gene ID were extracted from such hits using Ensembl biomart, and added to the annotation. In parallel, sequences of each feature were used as a blastn query to the genbank nr database; best blast hits were then extracted to provide a complementary annotation, especially valuable for fish specific genes, or for families with multiple paralogues in fish. To reduce the redundancy, we considered that different probes matching the same hit reflected the expression of the same "gene"; the average of corresponding fold changes was computed and used in figure 2, figure 5 and table S4.

*QPCR validation of micro array results*

First-strand cDNA was synthetized from total RNA (1 µg) using a QuantiTech Reverse Transcription Kit (QIAGEN), with an integrated genomic DNA elimination step, as per the manufacturer’s guidelines. The cDNA samples were diluted 100-fold with molecular biology grade water and stored at -20°C until qPCR analyses were performed using an Mx3005P qPCR System (Agilent Technologies) with SYBR Green I dye chemistry. Reactions (15µl volume) contained 5µl of cDNA (corresponding to 2.5ng of input total RNA), 500nM sense/antisense primers and 7.5µl Brilliant III Ultra-Fast SYBR Green (Agilent Technologies). The thermal cycling conditions were 1 cycle of 95°C for 3 minutes, followed by 40 cycles of 95°C for 20 seconds and 64°C for 20 seconds. Dissociation analysis (thermal gradient from 55°C to 95°C) was performed to confirm the amplification of a single product. Assays were performed in technical duplicate within 96-well plates (Agilent Technologies) and included no-template controls in duplicate (cDNA replaced with water).

The efficiency of each qPCR assay was determined using LinRegPCR following the author’s recommendations (Ruijter et al., 2009). Baseline corrected cycle threshold (Cq) values (threshold standardized across plates) were imported into GenEx (MultiD Analyses AB) and corrected for differences in efficiency. Within Genex, NormFinder (Andersen at al., 2004) and GeNorm (Vandesompele et al. 2002) were used to analyse the stability and suitability of five reference genes: Ribosomal Protein L4 (*RpL4*), Ribosomal Protein S13 (*RpS13*), Ribosomal Protein S29 (*RpS29*), β Actin (*ACTB*), and Elongation Factor 1α (*EF-1α*). The analysis revealed *RpL4* and e*EF-1α* to be the most stable pair of reference genes across all samples, hence, these genes were used to normalize Cq values of each experimental gene, which were converted to a scale of relative expression that was quantitative across different genes.

Pathogen loads for *A. salmonicida* and VHSV were established by comparing disease challenged animals to controls using the prodcedure described above, and from equal amounts of total RNA. Assays were performed in technical triplicates within 96-well plates (Agilent Technologies), included no-template controls in triplicates (cDNA replaced with water).
